# Radiographic occult cerebellar germinoma presenting with progressive ataxia and cranial nerve palsy

**DOI:** 10.1186/s12883-015-0516-9

**Published:** 2016-01-12

**Authors:** Noriaki Minami, Kazuhiro Tanaka, Hidehito Kimura, Takanori Hirose, Tatsuya Mori, Masahiro Maeyama, Hiroaki Sekiya, Takeshi Uenaka, Satoshi Nakamizo, Hiroaki Nagashima, Katsu Mizukawa, Tomoo Itoh, Takashi Sasayama, Eiji Kohmura

**Affiliations:** Department of Neurosurgery, Kobe University Graduate School of Medicine, Kusunoki-cho 7-5-2, Chuo-ku, Kobe, Hyogo 650-0017 Japan; Department of Pathology for Regional Communication, Kobe University Hospital, Kobe, Japan; Department of Neurology, Kobe University Graduate School of Medicine, Kobe, Japan; Department of Diagnostic Pathology, Kobe University Hospital, Kobe, Japan

**Keywords:** Cerebellar germinoma, Occult germinoma, T2 star-weighted image, Susceptibility-weighted image, Hypointensity

## Abstract

**Background:**

Although the usefulness of susceptibility-weighted imaging (SWI) for detecting basal ganglia germinoma has been reported, the technique is not widely used. We recently encountered an unusual case of primary cerebellar germinoma, presenting with progressive ataxia and cranial nerve palsy, characterized by gradually enlarging low-intensity lesions visible with both T2*-weighted imaging (T2*WI), which were the key to the diagnosis.

**Case presentation:**

A 30-year-old man was referred to our hospital because of slowly progressive dizziness and mild ataxia. Magnetic resonance imaging (MRI) revealed a small, low-intensity spot in the left cerebellar peduncle on the T2*WI and SWI without enhancement. Cerebral angiography revealed no vascular abnormality. The serum α-fetoprotein value was normal. A steroid-pulse was administered as a therapeutic and diagnostic trial, but the symptoms improved little. The patient was discharged from the hospital but soon developed brainstem dysfunction, characterized by dyspnea or hiccups, and he was readmitted. T2*WI imaging revealed expanded and extended spotty lesions in the cerebellum and brainstem, which had not enhanced with contrast agent previously. Targeted stereotactic biopsy of the newly enhanced cerebellar lesion was performed; histopathological examination of the tissue revealed pure germinoma. Serum and cerebral spinal fluid values of beta-human chorionic gonadotropin were not significantly elevated. Chemotherapy with carboplatin and etoposide was initiated. The enhanced lesion disappeared promptly, but the patient continued to require assisted automatic ventilation because of paralysis of respiratory muscles.

**Conclusions:**

We conclude that enlarging low-intensity lesions on T2*WI and SWI may be a reliable clue to the diagnosis of germinomas, irrespective of their location, even without enhancement. Biopsy of the tumor at an early stage is the only way to make the diagnosis conclusively and enable prompt start of treatment.

**Electronic supplementary material:**

The online version of this article (doi:10.1186/s12883-015-0516-9) contains supplementary material, which is available to authorized users.

## Background

Intracranial germ cell tumors (GCT) are rare, comprising only 0.6–2.8 % of all intracranial tumors [1–3]. About 40 % of GCTs are germinomas [4]. They may arise from basal ganglia, cerebellum, and brainstem. Radiographically, these tumors typically show isointense signals by T1 or T2-weighted MRI but are revealed as homogeneously enhanced lesions after contrast administration. Therefore, without enhancement by a contrast agent, GCTs are quite difficult to diagnose when present in atypical locations. Here, we report a rare case of cerebellar germinoma presenting with progressive ataxia and cranial nerve palsy, characterized by gradually enlarging multiple low-intensity lesions visible with both T2*-weighted imaging (T2*WI) and susceptibility weighted imaging (SWI).

## Case presentation

**Fig. 1:**
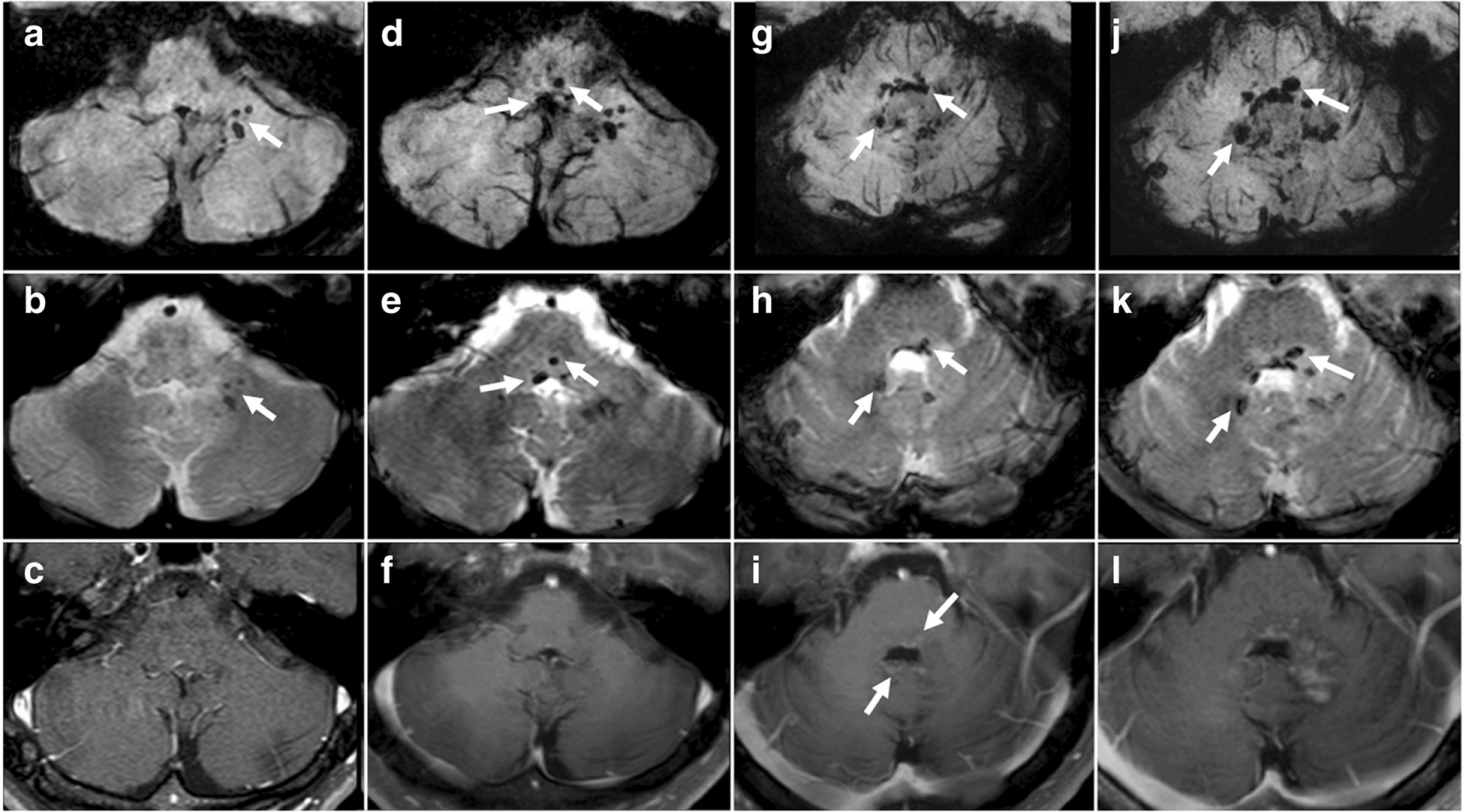
**a** SWI on the first admission revealed low-signal intensity spotty lesions in the left cerebellar peduncle (arrow). **b** T2*WI on the first admission. Findings similar to those in Fig. 1a are present (arrow). **c** Contrast-enhanced MRI on the first admission showed no apparently enhanced lesions, including in the cerebellar peduncle. **d** SWI obtained three months after the first admission revealed de novo low-intensity spots in the medulla oblongata. **e** T2*WI obtained three months after the first admission. Findings are to those in Fig. 1d. **f** Contrast-enhanced MRI obtained three months after the first admission showed no apparently enhanced lesions. **g** SWI obtained one year after the first admission revealed spreading hypointensity- lesions adjacent to the fourth ventricle (arrows). **h** T2*WI obtained one year after the first admission. Findings similar to those in Fig. 1g are present. **i** Contrast-enhanced MRI obtained one year after the first admission showed a slightly enhanced lesion around the fourth ventricle. **j** SWI on the second admission revealed expanding lesions of the pons and right cerebellar peduncle (arrows). **k** T2*WI on the second admission revealed findings similar to those in Fig. 1k. **l** Contract-enhanced MRI on the second admission showed an expanded enhanced lesion around the fourth ventricle

A 30-year-old male was referred to our hospital because of symptoms of slowly progressive dizziness and mild ataxia. He had been in good health until three years earlier except for the past medical history of hepatitis B, when he presented with transient dizziness without any abnormal findings on MRI (see Additional file 1: Figure S1). Two years previously, he began experiencing double vision, ptosis of the right eyelid, and left upper limb ataxia, which gradually worsened. He was first admitted to the department of neurology in our hospital because degenerative neurological disorder or other autoimmune diseases were suspected. Hematological examinations were normal. Conventional MRI revealed no specific findings, with the exception of three small low-intensity spots located in the left cerebellum on T2*-weighted image (T2*WI) and susceptibility-weighted image (SWI) that were not enhanced by gadolinium (Fig. 1a, b, c). Cerebral angiography showed no vascular abnormality. Because of his history of chronic hepatitis B, the serum α-fetoprotein had been measured several times, with negative results. Cerebrospinal fluid examinations were normal, except for an oligoclonal band. Autoimmune diseases or degenerative neurological disorders were suspected, but despite the initiation of steroid pulse therapy, the patient’s symptoms remained unchanged. Three months later, follow-up MRI studies revealed enlarged low-intensity lesions in the cerebellum and newly visible lesions in the brainstem with T2*WI and SWI (Fig. 1d, e, f). Nine months later, low intensity lesions were observed in the right cerebellar hemisphere, vermis, and dorsal surface of the brainstem with T2*WI and SWI (Fig. 1g, h, i). At this time, newly enhanced lesions surrounding the fourth ventricle were identified. However, a biopsy was not performed because the lesions were deeply seated and the patient’s clinical symptoms were stable.

**Fig. 2:**
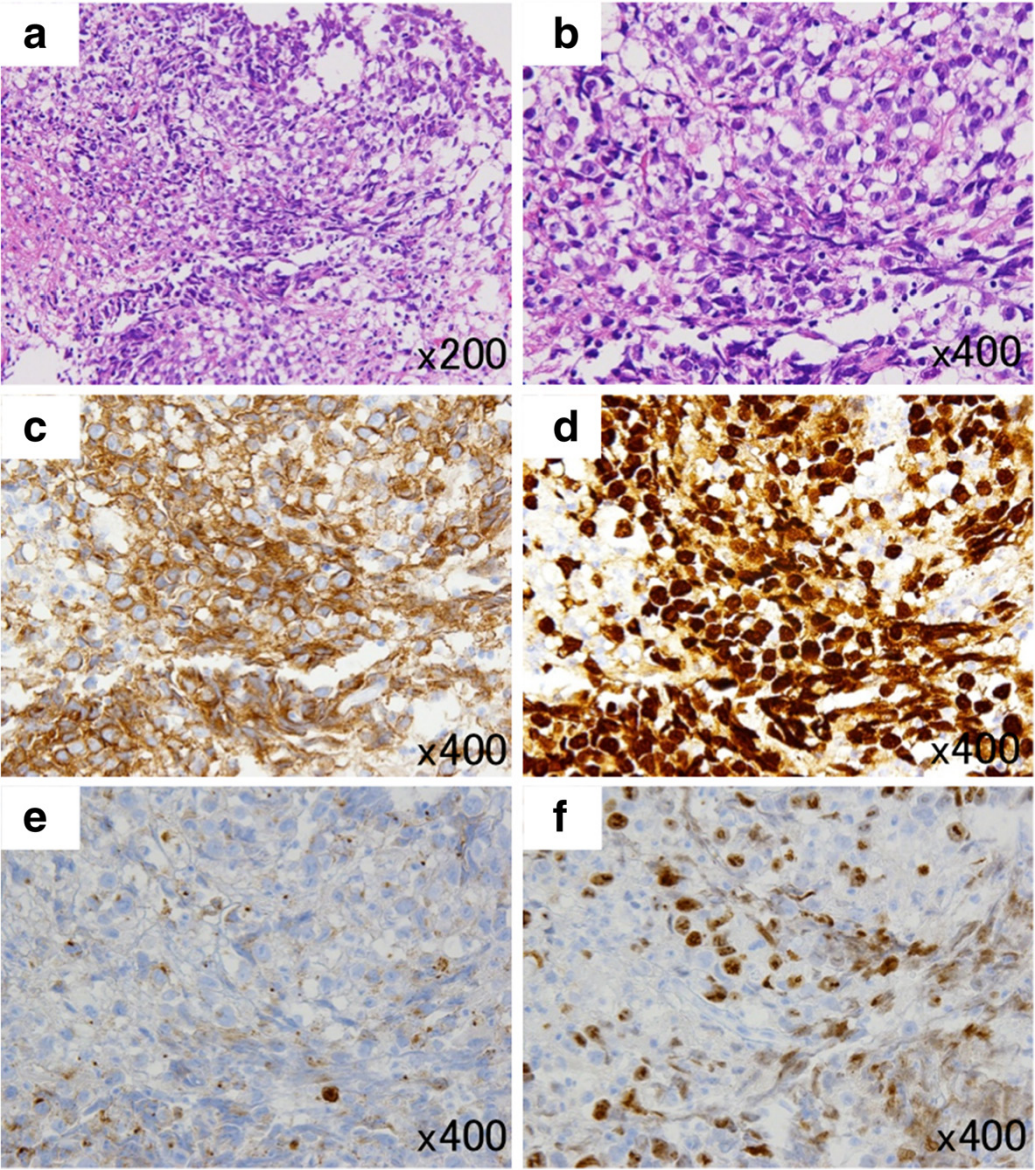
Histological findings of the tumor **a** and **b**; **h**-**e** stain of the tumor. The tumor is composed of sheets and lobules of large and pale cells with often indistinct cell membranes and somewhat vacuolated cytoplasm. Locally, lymphocytic infiltration is present. **c**-**f** Immunohistochemically, tumor cells are positive for c-Kit (**c**) and strongly positive for SALL4 (**d**). Also, tumor cells are weakly positive for PLAP (**e**). The Ki-67 labeling index is high in the tumor cells (**f**). (Original magnification; A × 200, B-F × 400)

Two months later, the patient was re-admitted with persistent hiccups, gait disturbance, and dysphagia. He became dyspneic and was intubated and ventilated. Alpha-fetoprotein and human chorionic gonadotropin β-subunit values were normal in serum and cerebral spinal fluid. MRI studies revealed mildly enhanced lesions surrounding the 4th ventricle and the left cerebellar hemisphere. With the use of T2*WI and SWI, low-intensity lesions were observed to be increased and expanded in bilateral cerebella and the dorsal brainstem. (Fig. 1j, k, l). A stereotactic biopsy was performed, targeting the newly enhanced lesion in the left cerebellum; histopathological examination revealed large and round neoplastic cells with lymphocytic infiltration suggestive of a two-cell pattern (Fig. 2a, b). The tumor cells tested immunopositive for c-Kit (Fig. 2c), SALL4 (Fig. 2d), and PLAP (Fig. 2e), characteristic of germinoma. Ki-67 labeling index was high (Fig. 2f). Soon after the pathological diagnosis was made, the patient underwent three cycles of chemotherapy with carboplatin (450 mg/m^2^, day1) and etoposide (150 mg/m^2^, day1-3). The enhanced lesions disappeared promptly, but low-intensity lesions remained visible by T2*WI and SWI. Radiation therapy was difficult to perform because of the patient’s poor physical condition. With rehabilitation, he made slight progress, and he now lives in his own house, but he remains almost bedridden and requires tracheostomy and ventilator assist because of the damage of the brainstem and cerebellum.

## Discussion

Germinomas typically arise in regions adjacent to the third ventricle, such as the pineal body and neurohypophysis, but they may be present also in the basal ganglia. Germinomas originating in the cerebellum are quite rare [5–11] (see Additional file 2: Table S1); in one study, only three of 153 intracranial GCTs were located in the cerebellum [12]. It is not difficult to diagnose intracranial GCTs when they have a typical location and radiographic features. However, an intracranial GCT located in the basal ganglia often is difficult to detect because enhancement is usually slight or absent [2]. Recent reports have emphasized the effectiveness of SWI in early detection of basal ganglia GCTs [13]. SWI can be used to detect blood products and accumulation of biologic metal, thereby providing additional information in evaluation of brain tumors. SWI may be useful also in the detection of intracranial GCTs, because this type of tumor is characterized by a high incidence of intra-tumor micro bleeds. Since T2*WI is also extremely sensitive for detecting micro bleeds in the brain, both techniques are considered useful for early detection of intracranial GCTs [14].

In the present case, low-intensity spots were present in the left cerebellar hemisphere with both T2*WI and SWI at early stages of tumor growth, when the tumor showed no enhancement after gadolinium administration. To our knowledge, this is the first report describing low-signal intensity lesions on T2*WI without any enhancement. Interestingly, low-signal lesions on T2*WI and SWI were found to concomitantly expand with tumor progression. Although having not been performed in our case, ^11^C-methionine positron emission topography provides valuable information for making the diagnosis of germinoma, and it is useful also for evaluating treatment effects and monitoring for tumor recurrence [4, 15].

Since intracranial GCTs are extremely sensitive to radiation, the possibility remains that our patient’s tumor became temporarily invisible by conventional MRI techniques due to the radiation exposure of the CT scan. Since diagnostic CT scans are required in clinical practice, the patient had received a CT scan prior to the initiation of the MRI studies. Although conventional MRI showed no unremarkable findings, low-signal lesions were observed with T2*WI and SWI, suggesting that low-signal lesions may not be affected by radiation and may be useful in detecting intracranial GCTs after radiation exposure associated with CT. This view is supported by the fact that the low-intensity lesions remained visible after chemotherapy, while gadolinium-enhanced regions disappeared.

The symptoms of intracranial GCTs depend on tumor location, and typical symptoms include hydrocephalus, diabetes insipidus, hypopituitarism, hemiparesis, or higher cognitive dysfunction [16]. Intracranial GCTs located near the fourth ventricle are also known to present with trigeminal, abducens, and facial nerve paresis and cerebellar signs [17]. In the present case, the patient presented with slowly progressive ataxia, followed by cranial nerve palsy and brainstem dysfunction, such as difficulty breathing. In such situations, patients are more likely to be consulted by neurologists or general physicians, because neurodegenerative diseases, infectious diseases, or autoimmune diseases are considered to be more likely. Therefore, not only neurosurgeons but neurologists, neuroradiologists, and general physicians should know that CNS germinomas could have the radiographic findings and symptoms described above. Although measuring the serum alpha-fetoprotein and human chorionic gonadtropin is important, they are often negative in pure germinomas, as in our case. The only test that conclusively makes the diagnosis is biopsy, and it should be performed without hesitation if the symptoms are progressive and if there are any abnormal lesions on MRI. Our study suggests that hypointensity changes in T2*WI and SWI are helpful for making the early diagnosis of intracranial GCTs arising in atypical locations.

## Conclusions

Enlarging, low-intensity lesions on T2*WI and SWI may be a reliable clue to the diagnosis of germinomas, irrespective of their location. Biopsy at the early stage is the only way to conclusively make the diagnosis and lead to early treatment.

## Consent

Written informed consent was obtained from the patient’s mother for publication of this case report and accompanying images. A copy of the written consent is available for review by the editor of this journal.

## Additional files

Additional file 1: Figure S1. The initial T2WI, FLAIR, and sagital T1WI image of the patient. No abnormal findings were detected on these sequences. (TIFF 3528 kb) Additional file 2: Table S1. Previously reported cases of cerebellar GCTs. (XLSX 10 kb)

## Abbreviations

GCT: Germ cell tumors
T2*WI T2*-weighted imaging: SWI: Susceptibility-weighted image
MRI: Magnetic resonance imaging

## References

[1] Committee of Brain Tumor Registry of Japan. Report of Brain Tumor Registry of Japan (1969–1996). Neurologia medico-chirurgica 2003, 43 Suppl:i-vii, 1–111 14705327

[2] Fujimaki T (2009). Central nervous system germ cell tumors: classification, clinical features, and treatment with a historical overview. J Child Neurol.

[3] Surawicz TS, McCarthy BJ, Kupelian V, Jukich PJ, Bruner JM, Davis FG (1999). Descriptive epidemiology of primary brain and CNS tumors: results from the Central Brain Tumor Registry of the United States, 1990–1994. Neuro Oncol.

[4] Lee J, Lee BL, Yoo KH, Sung KW, Koo HH, Lee SJ (2009). Atypical basal ganglia germinoma presenting as cerebral hemiatrophy: diagnosis and follow-up with 11C-methionine positron emission tomography. Childs Nerv Syst.

[5] El Abbadi N, Maaqili MR, Arkha Y, Amarti A, Bellakhdar F (2002). Cerebellar primary germinoma. Case report. Neurochirurgie.

[6] Evanson EJ, Lewis PD, Colquhoun IR (1997). Primary germinoma of the posterior cranial fossa: a case report. Neuroradiology.

[7] Maiuri F, Cappabianca P, Del Basso De Caro M, Esposito F, de Divitiis E (2004). Primary cerebellar germinomas of the posterior fossa. Br J Neurosurg.

[8] Ng HK, Poon WS (1990). Primary germinoma of the posterior fossa with CSF and extracranial metastases. Br J Neurosurg.

[9] Ichikawa T, Hamazaki S, Sakai N, Otsuki Y, Wataya T, Kambara H (2011). Mixed germ cell tumor and hemangioblastoma in the cerebellum: report of a rare coexistence. Brain Tumor Pathol.

[10] Nakase H, Ohnishi H, Touho H, Karasawa J, Tsunoda S (1994). Cerebellar primary germ-cell tumor in a young boy. Brain Dev.

[11] Shenoy AS, Desai HM, Tyagi DK, Savant HV, Kavishwar VS, Balasubramaniam M (2014). Primary yolk sac tumor of the cerebellar vermis: a case report. Indian J Pathol Microbiol.

[12] Matsutani M, Sano K, Takakura K, Fujimaki T, Nakamura O, Funata N (1997). Primary intracranial germ cell tumors: a clinical analysis of 153 histologically verified cases. J Neurosurg.

[13] Lou X, Ma L, Wang FL, Tang ZP, Huang H, Cai YQ (2009). Susceptibility-weighted imaging in the diagnosis of early basal ganglia germinoma. AJNR Am J Neuroradiol.

[14] Chavhan GB, Babyn PS, Thomas B, Shroff MM, Haacke EM (2009). Principles, techniques, and applications of T2*-based MR imaging and its special applications. Radiographics.

[15] Okochi Y, Nihashi T, Fujii M, Kato K, Okada Y, Ando Y (2014). Clinical use of (11)C-methionine and (18)F-FDG-PET for germinoma in central nervous system. Ann Nucl Med.

[16] Rasalkar DD, Chu WC, Cheng FW, Paunipagar BK, Shing MK, Li CK (2010). Atypical location of germinoma in basal ganglia in adolescents: radiological features and treatment outcomes. Br J Radiol.

[17] Yoshida K, Nakao Y, Yamamoto T, Mori K, Maeda M (2003). Germinoma in the fourth ventricle. Acta Neurochir.

