# Supplementary figures and images for: Radiographic occult cerebellar germinoma presenting with progressive ataxia and cranial nerve palsy
Source: BMC Neurol. 2016 Jan 12;16:4. doi: 10.1186/s12883-015-0516-9 (PMC4709897; doi:10.1186/s12883-015-0516-9)

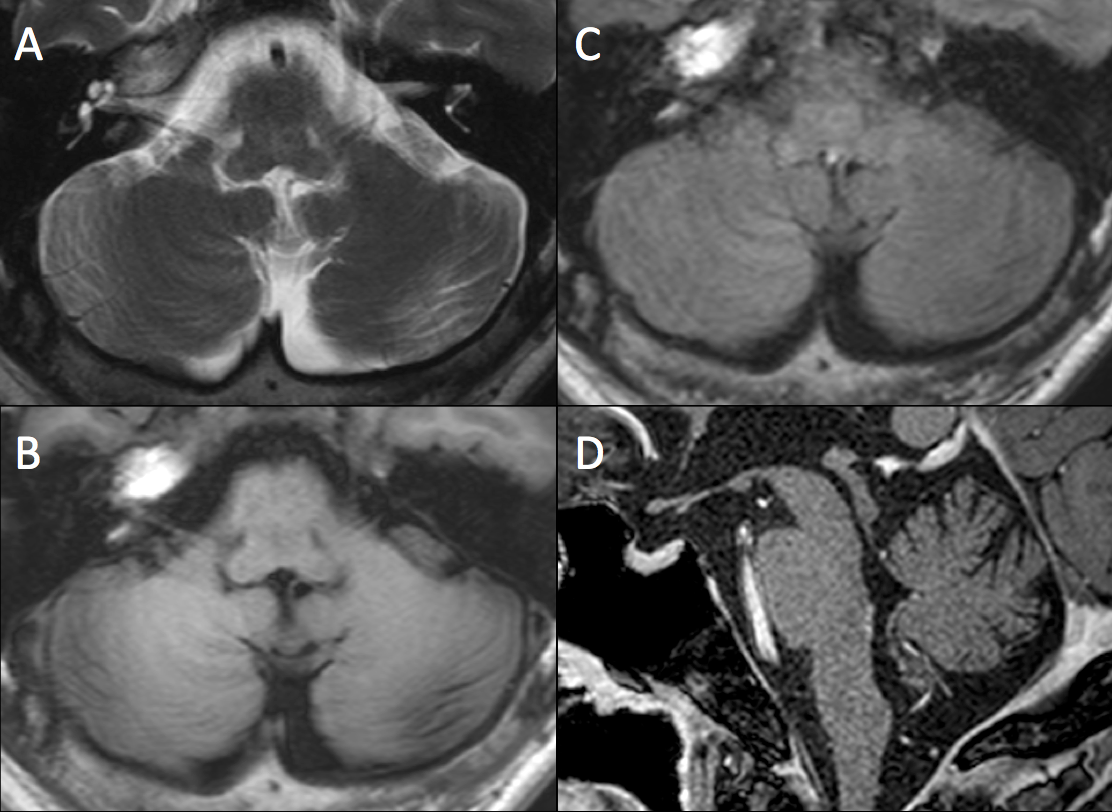

Supplement: Additional file 1: Figure S1. — The initial T2WI, FLAIR, and sagital T1WI image of the patient. No abnormal findings were detected on these sequences. (TIFF 3528 kb) [file 12883_2015_516_MOESM1_ESM.tiff]
